# Neural networks for action representation: a functional magnetic-resonance imaging and dynamic causal modeling study

**DOI:** 10.3389/fnhum.2012.00236

**Published:** 2012-08-14

**Authors:** Akihiro T. Sasaki, Takanori Kochiyama, Motoaki Sugiura, Hiroki C. Tanabe, Norihiro Sadato

**Affiliations:** ^1^Division of Cerebral Integration, Department of Cerebral Research, National Institute for Physiological SciencesOkazaki, Japan; ^2^Department of Physiological Sciences, The Graduate University for Advanced Studies (Sokendai)Okazaki, Japan; ^3^Research Fellow of the Japan Society for the Promotion of ScienceTokyo, Japan; ^4^The Hakubi Project, Primate Research Institute, Kyoto UniversityKyoto, Japan; ^5^Institute of Development, Aging and Cancer, Tohoku UniversitySendai, Japan; ^6^Biomedical Imaging Research Center, University of FukuiEiheiji, Japan

**Keywords:** cross-modal transformation, mirror system, network analysis, posterior parietal cortex, sensorimotor integration

## Abstract

Automatic mimicry is based on the tight linkage between motor and perception action representations in which internal models play a key role. Based on the anatomical connection, we hypothesized that the direct effective connectivity from the posterior superior temporal sulcus (pSTS) to the ventral premotor area (PMv) formed an inverse internal model, converting visual representation into a motor plan, and that reverse connectivity formed a forward internal model, converting the motor plan into a sensory outcome of action. To test this hypothesis, we employed dynamic causal-modeling analysis with functional magnetic-resonance imaging (fMRI). Twenty-four normal participants underwent a change-detection task involving two visually-presented balls that were either manually rotated by the investigator's right hand (“Hand”) or automatically rotated. The effective connectivity from the pSTS to the PMv was enhanced by hand observation and suppressed by execution, corresponding to the inverse model. Opposite effects were observed from the PMv to the pSTS, suggesting the forward model. Additionally, both execution and hand observation commonly enhanced the effective connectivity from the pSTS to the inferior parietal lobule (IPL), the IPL to the primary sensorimotor cortex (S/M1), the PMv to the IPL, and the PMv to the S/M1. Representation of the hand action therefore was implemented in the motor system including the S/M1. During hand observation, effective connectivity toward the pSTS was suppressed whereas that toward the PMv and S/M1 was enhanced. Thus, the action-representation network acted as a dynamic feedback-control system during action observation.

## Introduction

Automatic mimicry is the spontaneous copying of the low level, kinematic features of action (Hamilton, 2008). Preverbal children spontaneously mimic each other as a form of communication (Nadel, 2002). Typically developed adults unconsciously mimic each other's meaningless actions to facilitate social interaction (Chartrand and Bargh, 1999; Lakin and Chartrand, 2003). Automatic mimicry therefore appears to be an important basis for social interaction. The basis of the automatic mimicry is motor and perception action representations are tightly linked in such a way that perceiving another person's action activates the same representations as performing the action. For example, the execution of a grasping movement is facilitated by showing a picture of a hand in a matching posture (Craighero et al., 2002; Vogt et al., 2003). Conversely, executing an action while concurrently observing an incongruent action, as opposed to a congruent action, leads to slower responses (Brass and Heyes, 2005). This common coding allows humans to embody the behaviors of others and to imagine what it would be like to perform them (Barsalou et al., 2003). However, it is unclear how motor and perception action representations are linked to form either a common action representation or its neural substrates. Previously, it was argued that this common representation could be formed as an internal model through Hebbian associations trained during motor execution (Keysers and Perrett, 2004; Del Giudice et al., 2009). The internal model was originally conceptualized in the context of motor control (Wolpert et al., 2003). Skilled motor behavior relies on learning to control the body and to predict the consequences. Prediction turns motor commands into expected sensory consequences, whereas control turns desired consequences into motor commands. The neural processes underlying prediction and control are known as the forward and inverse internal models, respectively (Flanagan et al., 2003). The rationale behind the proposal by Keysers and Perrett (2004) was that motor control requires sensory feedback. Given that we continuously monitor our own actions through proprioception, somatosensation, vision, and audition, their sensory consequences are systematically and synchronously paired with motor commands. This predicts the emergence of Hebbian connections that link motor programs to sensory consequences (forward internal models), and sensory consequences to motor programs (inverse internal models).

One possible neural mechanism contributing to the action representation involves mirror neurons, which comprise a class of visuomotor neurons discovered in area F5 of the monkey ventral premotor cortex (PMv; di Pellegrino et al., 1992; Gallese et al., 1996; Rizzolatti et al., 1996), and subsequently reported in area PF of the posterior parietal cortex (PPC; Rizzolatti et al., 2001). Mirror neurons discharge when a monkey performs a particular action, and when it observes another monkey or human performing a similar action (for a review see Rizzolatti and Craighero, 2004). These primate frontoparietal areas and the superior temporal sulcus (STS) have been implicated in the capacity to associate observed actions with self actions, thus forming action representation. Keysers and Perrett (2004) proposed a physiologically plausible model of how the F5–PF–STS circuit, working through Hebbian learning, could associate observed actions with a monkey's own actions (i.e., mirror properties) and discriminate self actions from those of others. In macaque monkeys, the STS does not have a direct connection to the F5, and models have assumed that “the intermediate stage between STS and F5 appears to be represented by the inferior parietal lobule (IPL) and, in particular, by area PF that receives afferents from STS and is connected with F5c” (Rizzolatti and Luppino, 2001), without considering a direct interaction between the STS and the F5.

Although functional magnetic-resonance imaging (fMRI) studies have revealed that the visual perception of an action engages compatible activity in an observer's motor system (Gazzola and Keysers, 2009; Caspers et al., 2010; Molenberghs et al., 2012), current evidence for human mirror neurons is still controversial or inadequate (Dinstein et al., 2007). Hence, it was sometimes called putative mirror neuron system (pMNS), which includes the PMv, IPL, and posterior portion of the STS (pSTS; Schippers and Keysers, 2011). The pMNS has been suggested to host forward and inverse models that work together to allow the prediction of others' intentions and behaviors (Blakemore and Decety, 2001; Miall, 2003; Keysers and Perrett, 2004; Csibra and Gergely, 2007; Kilner et al., 2007a,b; Lamm et al., 2007; Gazzola and Keysers, 2009). In contrast to non-human primates, diffusion-tensor imaging (DTI) of the human brain has shown direct connections between middle temporal and inferior frontal areas (Catani et al., 2005; Rilling et al., 2008); their direct interaction should therefore be considered (Hamilton, 2008). Hamilton (2008) proposed the EP-M model, which divides the MNS into an indirect, parietal route for goal emulation and planning (EP), and a direct occipital-frontal route for mimicry (M).

Prompted by the EP-M model, we hypothesized that the direct connection from the STS to the PMv forms an inverse internal model, converting visual representation into a motor plan, and that reverse connections form a forward internal model, converting the motor plan into a sensory outcome of action. In this scenario, observation of others' actions without goal inferences should activate the inverse model represented by the information flow from the STS to the PMv, and the execution of action should activate the forward model represented by the information flow from the PMv to the STS.

To test this hypothesis, we used fMRI with dynamic causal modeling (DCM; Friston et al., 2003) to delineate the dynamics of the neural networks for action representation via effective connectivity, which is defined as the influence that one neural system exerts over another (Friston et al., 1994). Participants performed a change-detection task during fMRI, in which they were asked to identify changes in the rotation speed of balls that were either actively manipulated by the right hand of the investigator (“Hand”) or automatically rotated (“No-hand”). Participants either rotated (“Execution”) or did not rotate (“Observation”) two balls while viewing the stimuli, giving a 2 (Execution vs. Observation) × 2 (Hand vs. No hand) task design. The two factors constituted the experimental manipulation that modulated the effective connectivity among the sensori-motor regions, and directly modulated the activities of the STS and the PMv.

The task design minimized the effect of goal-directed action understanding and intention to imitate, thereby allowing evaluation of the neural substrates and the network dynamics of automatic mimicry (by means of the Hand effect). To consider the involvement of regions other than the pMNS, we included the areas activated by execution, such as the primary sensori-motor cortex (S/M1), as regions of interest (ROIs). Primary motor cortex excitability was shown to be modulated by the observation of action (Fadiga et al., 1995; Strafella and Paus, 2000; Maeda et al., 2001, 2002) using motor evoked potentials induced by transcranial magnetic stimulation (TMS). A previous positron-emission tomography (PET) study showed the involvement of the S/M1 during the perception of hand action (Grezes et al., 1998). The S/M1 is therefore expected to be involved in action representation (Gazzola and Keysers, 2009). However, little is known about the relationship between the pMNS and the M1 in terms of action representation (Fadiga et al., 2005; Kilner and Frith, 2007). Finally, we also employed electromyography (EMG) of the right hand, which is an accurate and implicit measure of automatic mimicry (McIntosh et al., 2006).

## Materials and methods

### Participants

The study group comprised 24 healthy volunteers (19 males and five females; mean age = 26.7 years; standard deviation [SD] = 4.46). All of the participants had normal or correct-to-normal visual acuity, and were right handed according to the Edinburgh handedness inventory (Oldfield, 1971). The protocol was approved by the Ethical Committee of the National Institute for Physiological Sciences, Japan. All of the participants gave written informed consent for involvement in the study.

### Experimental design

The task involved two-ball rotation with the right hand (Matsumura et al., 2004). All of the participants successfully acquired this motor skill in a clockwise direction through pre-scanning training. The direction of rotation of the two visually-presented balls was either clockwise or counter-clockwise. To focus their attention, participants were required to detect the speed of change of two rotating balls presented on a screen. This was intended to minimize the confounding effects of action understanding or intent to imitate, and thereby to clarify the specific neural activities stimulated by the action representation of ball rotation.

### Stimulus preparation

#### Original video clip

An original video clip was recorded in which a ball was rotated once by the investigator (Akihiro T. Sasaki) with his right hand, viewed from above against a black cloth background, using a video camera (Sony Handy Cam; Sony, Tokyo, Japan). The original video clip was edited using a time interval of either 1 s per rotation (equivalent to 60 revolutions per min [rpm]) or 0.75 s per rotation (80 rpm) by Adobe-Premiere software (Adobe System Inc., San Jose, CA).

#### Rotation by hand

**Figure 1:**
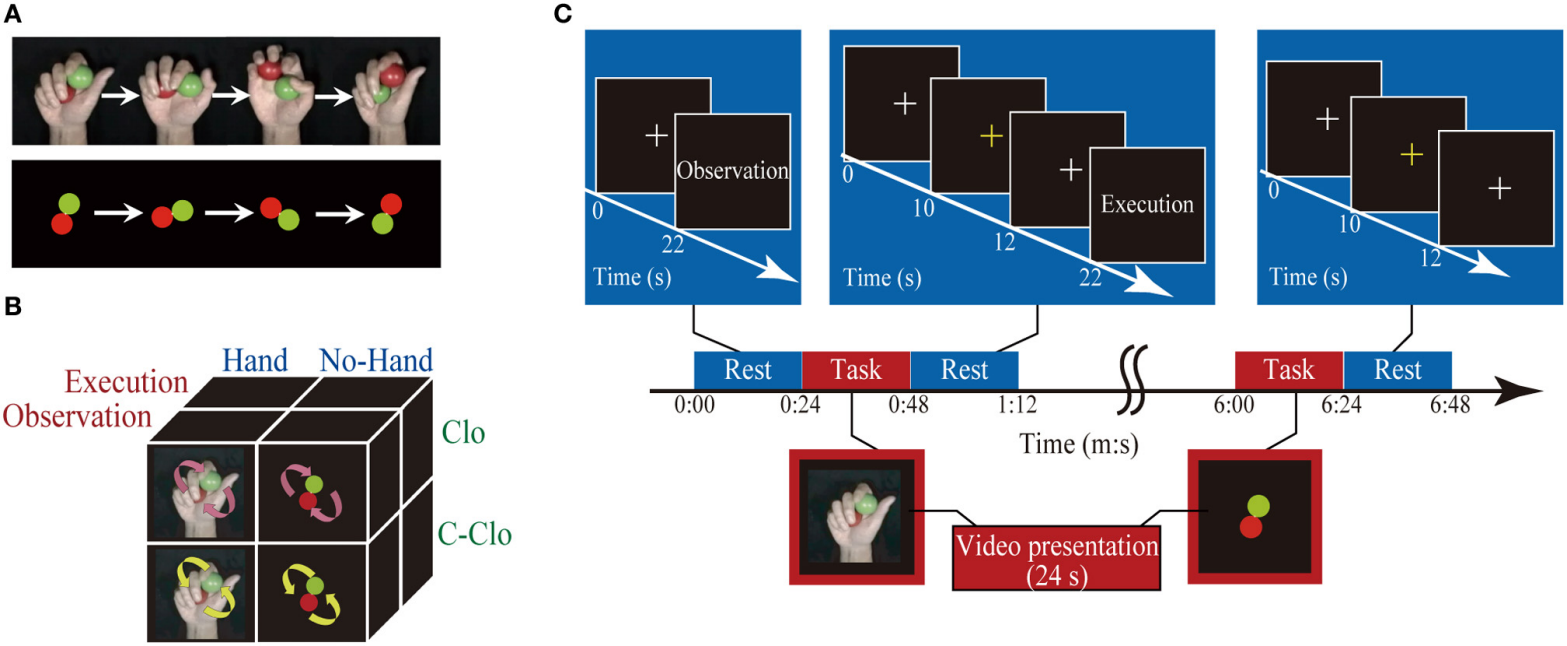
**Schematic diagram of an experimental run. (A)** Sample frames of visual stimuli: clockwise rotation of two balls with a hand (upper row) and without a hand. **(B)** Schema of factorial design: arrows indicate the direction of ball rotation. **(C)** Schematic diagram of an experimental run: the rest and task conditions, each 24 s in duration, were presented alternately. The first rest epoch comprised presentation of a white fixation crosshair, and instruction on the subsequent task (Execution or Observation) presentation. Rest epochs, except for the first and last, comprised presentation of a fixation crosshair, a yellow fixation crosshair response cue that prompted the participant to state the number of changes in speed in the preceding task condition, and an instruction (“Execution” or “Observation”). The last rest epoch comprised a white fixation crosshair and response cue. During the task epochs, one of the visual stimuli was presented for 24 s. The numbers under the arrows indicate the time.

The video clips were concatenated to generate footage of two balls being rotated in a clockwise direction by a right hand for 24 s (Figure 1A), in which the speed was either kept constant or changed once or twice. We prepared a set of video clips in which the baseline speed of 60 rpm was altered to 80 rpm when indicated. In the “NONE” condition, the speed of ball rotation was kept constant. In the “ONCE” condition, the speed of ball rotation was changed to 80 rpm at 6, 12, or 18 s after the start of the clip, and was kept at the higher speed until the end of the clip. In the “TWICE” condition, the speed of ball rotation was changed to 80 rpm at 6 or 12 s after the start of the clip, and was returned to 60 rpm at 18 s. We also prepared a similar set of video clips in which the ball was rotated in a clockwise direction but the baseline frequency was 80 rpm and the altered frequency was 60 rpm. Additionally, we prepared an equivalent set of video clips in which the ball was rotated by hand in the counter-clockwise direction, by reversing the video clips for the clockwise set.

#### Automatic rotation

An original video clip of automatic ball rotation without a hand (Figure 1A) was produced using Illustrator software (Adobe System Inc.) and Windows XP Movie Maker (Microsoft Corp., Redmond, WA). Red and green filled circles were generated, jittered by 4° to generate 90 images, and combined to create an animation of one rotation. The speed of the original animation was adjusted to create similar sets of video clips to those described above for rotation by hand, using an identical sequence of ball rotation.

### Pre-scan training

Before the fMRI session, a single training session was conducted in an experimental room adjacent to the scanner room. The participants were instructed to lie on a bed in a supine position, and to rotate two balls (diameter = 4 cm for each; weight = 114 g for each; one red and one green) in a clockwise direction with their right palms as quickly as possible (Matsumura et al., 2004). The training session consisted of 20 rotation epochs, each of which was 30 s in duration, alternated with 19 rest epochs. A Sony Handy Cam was used to record the training session, and was positioned such that the participant's right hand was at the center of the imaging frame.

### Participant preparation

Inside the scanner, the participants were instructed to place their right hands, palm upwards, along the right side of their bodies, and to place their left hands over a box with four buttons (Resonance Technology, Inc., Northridge, CA). Two balls, identical to those used in the pre-fMRI session, were placed in the palm of each participant's right hand at the beginning of the session. Throughout the session, the participants were asked to fixate a small white crosshair at the center of the screen.

### Experimental settings

Stimulus presentation and response collection were performed using Presentation 0.90 software (Neurobehavioral Systems, CA) implemented on a personal computer (Dimension 9100; Dell Computer Co., TX). A liquid crystal display (LCD) projector (DLA-M200L; Victor, Yokohama, Japan) located outside and behind the scanner projected the stimuli through another waveguide onto a translucent screen, which the participants viewed via a mirror attached to the head coil of the scanner. A video camera with a sampling rate of 30 frames/s (fps) was positioned above the MRI scanner such that the participant's right hand was at the center of the imaging frame. The performance of the right hand and the presented stimuli were projected onto the same monitor using a splitter, and were recorded simultaneously.

To confirm that all of the participants successfully conducted the Observation or Execution task, the EMG signal was recorded from the right flexor carpi ulnaris (FCU) and the extensor digitorum communis (EDC) muscles with disposable silver (Ag)-silver chloride (AgCl) surface electrodes (F-150; Nihon Kohden, Tokyo, Japan). The EMG activities were amplified 500 times and high-pass filtered (10 Hz; EMG-025; Harada Electronics Industry Ltd, Sapporo, Japan). The EMG data were recorded on a personal computer for subsequent off-line analyses via an analog-to-digital (A/D) converter (ML880, Powerlab 16/30; ADInstruments, Pty Ltd., Castle Hill, Australia).

### fMRI design

The fMRI session had a block design and comprised six runs. In a single run, eight task epochs and nine rest epochs, each of which was 24 s in duration, were presented in an alternating pattern beginning with a rest epoch. During the task epochs, each participant had to execute the ball rotation or remain inactive while viewing four types of visual clip, in which two balls were rotated in a clockwise or counter-clockwise direction, with or without a hand. Each run contained eight task conditions as follows: (Execution, Observation) × (Hand, No-hand) × (Clockwise, Counter-clockwise) (Figure 1B). The order of the task conditions was pseudo-randomized across the runs.

During the first rest epoch, a white fixation crosshair was presented at the center of the screen for 20 s, followed by an instruction cue that indicated whether the next task condition was Observation or Execution. During Execution epochs, the participants were required to observe visual stimuli and count the number of speed changes of the two rotating balls, while executing two-ball rotation in the clockwise (learned) direction with their right hands at the same speed. During the subsequent rest epoch, the color of the fixation crosshair was changed to yellow for 2 s, prompting the participants to use their left fingers to press the button that corresponded to the number of speed changes observed during the preceding task epoch, as follows: the index finger was used for “no change,” the middle finger for “once,” and the ring finger for “twice.” In the Execution/Hand/Clockwise condition, video clips of Hand/Clockwise rotation that started at either 60 rpm or 80 rpm were presented. The Execution/Hand/Counter-clockwise condition was similar, except that the video clips ran in reverse so that the direction of the two-ball rotation was counter-clockwise. The Execution/No-hand condition was similar to the Execution/Hand condition, except that the video clips showed clockwise two-ball rotation without a hand. The Observation epochs were similar to the Execution epochs, except that participants were instructed not to move their right hands. The final rest epoch was similar to the second-to-eighth rest epochs, except for the absence of an instruction cue (Figure 1C).

### MRI data acquisition

All images were acquired using a 3T MR scanner (Allegra; Siemens, Erlangen, Germany). For functional imaging during the sessions, an ascending T2^*^-weighted gradient-echo echo-planar imaging (EPI) procedure was used to produce 3-mm-thick transaxial slices (36 in total) with a 0.45-mm gap covering the entire cerebral and cerebellar cortices (repetition time [TR] = 3000 ms; echo time [TE] = 30 ms; flip angle [FA] = 80°; field of view [FOV] = 192 mm; 64 × 64 matrix; voxel dimensions = 3.0 × 3.0 × 4.0 mm; phase oversampling = 50%). The acquisition time (TA) was set at 2400 ms, so as to obtain a 600-ms “silent period” without any magnetic-field gradient or radio-frequency pulse. This was intended to reduce the artifacts in the EMG recording during the fMRI run. In total, 816 volumes (136 volumes per run) were acquired. For anatomical imaging, high-resolution whole-brain MR images were also obtained using a T1-weighted three-dimensional (3D) magnetization-prepared rapid-acquisition gradient echo (MPRAGE) sequence (TR = 2500 ms; TE = 4.38; FA = 8°; FOV = 230 mm; one slab; 192 slices per slab; voxel dimensions = 0.9 × 0.9 × 1.0 mm).

### Data analysis

#### Behavioral data analysis

The reaction time and the percentage of correct responses for the number of changes in speed of two-ball rotation were measured. A one-sample *t*-test was conducted in each condition to compare participants' performance against an expected chance level of 33.3%.

#### EMG analysis

The EMG recordings were rectified and integrated for every 600-ms silent period of volume acquisition for the MRI data. In total, 48 silent periods were used for each experimental condition, and 432 were used for the rest condition. The EMG recordings of both the FCU and the EDC muscles showed clear phasic-activity patterns during the ball-rotation sessions, so the averaged values were calculated. To normalize inter-individual variation, the integrated EMG values were transformed to Fisher's *z*-scores. The task-related EMG activation was calculated by subtracting the EMG signal at rest from that during the task.

#### Imaging data analysis

***Preprocessing***. The first three volumes of each run were discarded for stabilization of the magnetization, and the remaining 133 volumes per run (a total of 798 volumes per participant for six runs) were used for the analysis. The data were analyzed using statistical parametric mapping (SPM8; Wellcome Trust Centre for Neuroimaging, London, UK; Friston, 2007) implemented in MATLAB (Mathworks, Natick, MA). Following realignment and slice-timing correction, all of the images were linearly and non-linearly transformed into an EPI template that was already fitted to a standard stereotaxic space defined by the Montreal Neurological Institute (MNI; Friston, 2007). The spatially normalized EPI images were filtered using a Gaussian kernel of 8 mm full-width at half maximum (FWHM) in the *x*, *y*, and *z* axes. T1 anatomical images were also normalized to a standard T1 template image.

***Statistical analysis***. We used random-effects analysis for the significantly activated voxels at the population level (Friston, 2007). Initially, we performed a single-participant analysis. The individual task-related activity was evaluated using a general linear model (Friston, 2007). The signal time-course of each participant was modeled with a boxcar function convolved with a hemodynamic-response function, a high-pass filter (with a cut-off period of 128 s), and session effects. For each run, we included eight regressors of each task condition, and one regressor for the timing of the response cue. Serial autocorrelation of the fMRI time series was modeled using a first-order autoregressive model. The resulting set of voxel values for each comparison constituted a statistical parametric map of the *t* statistic [SPM {*t*}].

The weighted sum of the parameters estimated in the individual analyses consisted of “contrast” images, which were used for the group analyses. The contrast images obtained from each individual analysis represented the normalized increment of the fMRI signal for each participant. The contrast images of all eight task conditions were entered into a factorial model for three-way (2 × 2 × 2) analysis of variance (ANOVA). The resulting set for each contrast constituted the SPM {*t*}, focusing on the main effects of Execution and Hand observation, and their interaction, as the learned direction was not the main concern of this study (Table 1). The statistical threshold was set at *p* < 0.05 with correction of the family-wise Type I error (FWE) at the voxel level (Friston, 2007).

**Table 1:** **Predefined contrasts for second-level analysis**.

**Task condition**	**Execution**				**Observation**			
**Visual input from screen**	**Hand**		**No-hand**		**Hand**		**No-hand**	
**Learned direction**	**Clo**	**C-Clo**	**Clo**	**C-Clo**	**Clo**	**C-Clo**	**Clo**	**C-Clo**
Execution	1	1	1	1	−1	−1	−1	−1
Hand	1	1	−1	−1	1	1	−1	−1
Execution × Hand	−1	−1	1	1	1	1	−1	−1

Clo, clockwise; C-Clo, counter-clockwise.

#### DCM

***Evaluation of effective connectivity***. DCM is based on a bilinear model of neural population dynamics that is combined with a hemodynamic model describing the transformation of neural activity into a measured blood oxygen level-dependent (BOLD) response (Friston et al., 2003). The aim of DCM is to estimate parameters at the neuronal level, such that the modeled BOLD signals are maximally similar to the experimentally measured BOLD signals. DCM for fMRI combines the neural dynamics model with an experimentally validated hemodynamic forward model (Buxton et al., 1998), which describes the transformation of neuronal activity into a BOLD response. The combined neural and hemodynamic parameter set is estimated from the measured BOLD data, using a fully Bayesian approach with empirical (for hemodynamic) and conservative shrinkage (for neural) priors for the coupling parameters.

DCM assumes that the neural dynamics are driven by experimentally controlled external inputs that can enter the model either by eliciting responses through direct influences on specific regions or by modulating the coupling among regions (Stephan et al., 2007). Thus, at the neural level, the following three sets of parameters are estimated; the fixed (or baseline) connectivity among the regions in the absence of input; the change in connectivity induced by the inputs; and the strength of direct influences of input on neuronal activity (Stephan et al., 2007). Specifically, the dynamic causal model is an input state–output system with bilinear differential equations, as shown in Equation 1 (Friston et al., 2003).

(1) $$\frac{dz}{dt}=\left(A+\sum_{j=1}^{m}\;\mu_{j}B^{\left(j\right)}\right)z+Cu_{j}$$

Here, *z* is the state vector (with each state variable representing the population activity of one region in the model), *t* is continuous time, and *u*_*j*_ is the *j*th input to the modeled system (that is, some experimentally controlled manipulation). Equation (1) models the changes in the states (the neuronal population activities) by the known inputs. The inputs *u* correspond to designed causes. The outputs correspond to the observed BOLD time series of the selected ROIs. The parameters in a DCM denote the rate of change of neuronal activity (in Hz) in one area as induced by an input or by the output from another area, respectively. The *A* matrix contains the “baseline” or “fixed” connection strengths between the modeled regions, and the *B*^(1)^ … *B*^(*m*)^ matrices represent the context-dependent modulation of these connections. The *C* matrix represents the strengths of direct inputs to the modeled system.

***Hypothesis***. Our hypothesis was that during observation of another's hand action, the visual signal is input to the pSTS and then directly to the PMv, forming an inverse internal model that converts the visual representation into a motor plan. Considering its reciprocity (Wolpert et al., 2003), during the execution of the hand movement, the motor command is directly input to the PMv, and then to the pSTS, forming a forward internal model that converts the motor plan into a sensory outcome of action. In the present experiment, the direct inputs were all visual stimuli for the change-detection task (driving input), the motor execution by the participants, and the hand movement presented in the visual stimuli. The last two of these were also modulators of the connectivity. The analysis focused on the following issues: first, whether the Execution effect and Hand observation effect input to the PMv and pSTS, respectively; second, whether there was direct baseline connectivity between them; and third, how the system of interest was modulated by the Execution and Hand effects. In the DCM analysis, we initially addressed the first two questions in the model space, in order to select the best model, assuming that the modulation occurred in all existing connections. Using the best model, the context dependency of the modulation effect was then evaluated in the parameter space.

***Preprocessing for DCM***. To evaluate the effective connectivity using DCM, EPI images were preprocessed in the same manner as for imaging data analysis except for the smoothing process: normalized EPI images were filtered using a Gaussian kernel of 4-mm FWHM in the *x*, *y*, and *z* axes to increase the regional specificity. Images from six separate runs, each containing 133 time points, were concatenated as a single run to form a single time series with 798 time points for each individual.

For simplicity (Stephan et al., 2010), a new design matrix was created for DCM analysis that modeled three critical factors (change detection task, Execution, and Hand observation) and effects of no interest (six run effects and six realignment parameters to account for motion-related variance). A high-pass filter with a cut-off period of 128 s was also modeled to remove low-frequency signal drifts. A first-order autoregressive model was used to remove serial autocorrelation in the fMRI time series.

***System of interest***. *Definition of regions of interest (ROIs)*. The system of interest consisted of the following seven regions: the occipital pole (OP) as the cortical entry site receiving the cue for the change-detection task as driving input; the visual motion-sensitive middle temporal visual area (MT/V5); the pSTS that is sensitive to biological motion (Keysers and Perrett, 2004); the IPL (Buccino et al., 2004; Vogt et al., 2007) and the anterior part of the intraparietal sulcus (aIPS; Shmuelof and Zohary, 2005, 2006; Hamilton and Grafton, 2006) that were commonly activated by the execution and observation of hand movement; and the PMv and the S/M1, which showed a motor execution effect in the present study, as parts of the motor execution network. These regions fulfilled the minimum requirements for the system of interest in the present study, and comprised the simplest possible circuit diagram (Aertsen and Presl, 1991), because the major elements of the action representation were visual inputs, motion perception, and motor execution.

*Definition of coordinates and data extraction from ROIs.* We determined the coordinates of the ROIs in the S/M1, PMv, aIPS, IPL, pSTS, MT/V5, and OP based on group analysis (Table 2), because not all subjects showed local maxima close enough to the reference points obtained by group analysis. The ROI coordinates were calculated as the local maximum voxel depicted by the [Execution vs. Observation] contrast for the S/M1 and PMv, and as the conjunction of the [Execution vs. Observation], [Hand vs. No-hand], and [Execution × Hand interaction] contrasts for the aIPS and IPL. Because of the task characteristics, the distinction between M1 and S1 is difficult thus we designated the activation close to the central sulcus as S/M1. The coordinates of the IPL (*x*, *y*, *z*) = (−58, −18, 40) (Table 2) were located close to those of the postcentral sulcus (PoCS). A recent cytoarchitectonic map of the human IPL and its surrounding structures showed that area PFt of the IPL (Caspers et al., 2006, 2008) extends into the caudal wall of the PoCS, in the rostral wall of which area 2 is located (Grefkes et al., 2001).

**Table 2:** **ROIs for effective-connectivity analysis**.

**ROI**	**Coordinate**		
	***x***	***y***	***z***
OP	−22	−100	4
MT/V5	−50	−72	0
pSTS	−50	−64	4
aIPS	−38	−42	60
IPL	−56	−18	40
PMv/IFG	−58	6	28
S/M1	−40	−20	58

OP, occipital pole; pSTS, posterior portion of superior temporal sulcus; aIPS, anterior intraparietal sulcus; IPL, inferior parietal lobule; PMv/IFG, ventral premotor/inferior frontal gyrus; S/M1, primary sensorimotor area.

Because the [Hand vs. No-hand] contrast activated a large area of the occipitotemporal cortex, we determined the ROIs in the pSTS, MT/V5, and OP as follows. The pSTS has been reported to respond to point-light biological motion (Grossman and Blake, 2002; Grossman et al., 2010), the observation of hand action (Bonda et al., 1996), and hand observation (Molenberghs et al., 2010). By averaging the coordinates reported in previous biological motion studies (Bonda et al., 1996; Grossman and Blake, 2002; Grossman et al., 2010; Molenberghs et al., 2010; Table 3), we determined the reference coordinates of the pSTS, adjusted for the differences between the Talairach and MNI coordinates (http://imaging.mrc-cbu.cam.ac.uk/imaging/MniTalairach). Using the reference coordinates (*x* = −48, *y* = −60, *z* = 10), the ROI for the pSTS was determined as the local maximum voxel in the group data that showed a positive hand-observation effect and was nearest to the reference coordinates. The determined coordinates of the pSTS (−50, −64, 4) were close to the coordinates (−51, −63, 6) obtained by the hand presentation (Molenberghs et al., 2010). The ROI for the MT/V5 was determined as the local maximum in the group data with a hand-observation effect that was nearest to the reference coordinates (*x* = −45, *y* = −74, *z* = 2) reported by Dumoulin et al. (2000). The ROI coordinates of the OP were determined as the local maximum voxel in the group data that showed a positive average effect for all conditions. The ROI coordinates were determined identically among all participants. The ROI time-series data for each participant were extracted from voxels within a 4-mm radius centered on predefined ROI coordinates (Table 2) to increase the regional specificity. The data were adjusted for effects of no interest, high-pass filtered, and corrected for serial correlation.

**Table 3:** **Peak coordinates in left STS**.

**Authors**	**Talairach coordinates**			**MNI coordinates**			**Location**
	***x***	***y***	***z***	***x***	***y***	***z***	
Bonda et al. (1996)	−48	−61	17	−48.5	−63.7	15.1	Upper bank of caudal STS
Molenberghs et al. (2010)	−	−	−	−51	−63	6	STS
Grossman and Blake (2002)	−41.3	−52.8	11.8	−41.7	−55	9.9	pSTS
Grossman et al. (2010)	−51.8	−57.9	9.5	−52.3	−60.1	7.2	pSTS
	−48.2	−58	14.2	−48.7	−60.5	12.3	pSTS
Average	−47.3	−57.4	13.1	−48.4	−60.4	10.1	

MNI coordinates were converted from Talairach coordinates using a non-linear algorithm (http://imaging.mrc-cbu.cam.ac.uk/imaging/MniTalairach), except for those reported by Molenberghs et al. (2010). (p)STS, (posterior part of the) superior temporal sulcus.

**Figure 2:**
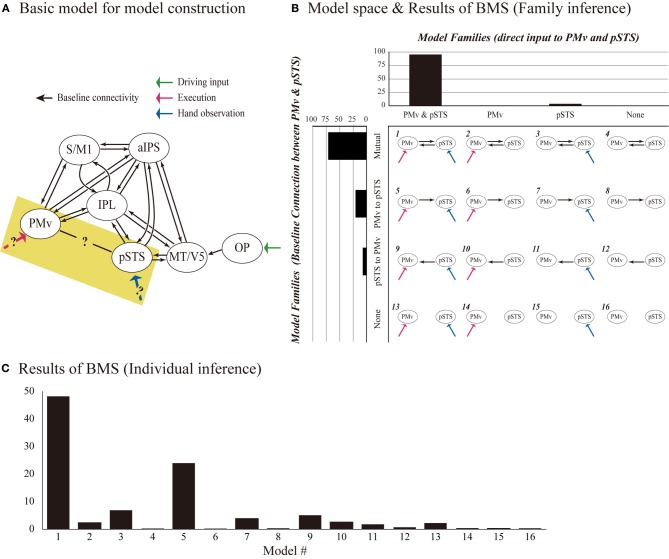
**Model definition and results of model selection. (A)** DCM models consist of seven ROIs (OP, MT/V5, pSTS, aIPS, IPL, PMv, and S/M1). All models had identical baseline connections (black arrows), except for that between the PMv and the pSTS, and had identical driving input to OP (green arrow). By manipulating the baseline connectivity between the PMv and the pSTS, and the direct inputs of execution to the PMv and hand observation to the pSTS (highlighted area), 16 DCM models were generated **(B)**. Rows indicate the presence/absence of direct connectivity from the PMv to the pSTS, and the pSTS to the PMv, and columns indicate the presence/absence of the input of Execution to the PMv and the input of Hand observation to the pSTS. We assumed that the two experimental factors (Execution and Hand observation) modulated all baseline connectivity. Upper bar graph shows the exceedance probability (%) of the four families partitioned by the patterns of the direct input to PMv and pSTS. Left bar graph indicates the exceedance probability (%) of four families based on the baseline connectivity between PMv and pSTS. **(C)** BMS-exceedance probability (%) resulted from 16 model comparison with individual inference. The number of model (horizontal axis) corresponds to the number shown in **(B)**.

*Definition of network models.* We modeled the connections among seven ROIs to test our hypothesis that there was direct, bidirectional connectivity between the PMv and the pSTS. We defined the models by manipulating the baseline connectivity, modulation, and direct inputs (Figure 2A). Initially, the baseline connectivity (A parameters) was assumed to be bidirectional among the execution related regions (the S/M1, IPL, PMv, and aIPS), and hand-observation related regions (pSTS, IPL, aIPS, and MT/V5) (Figure 2A). This was based on previous anatomical studies with non-human primates (for a review, see Rizzolatti and Matelli, 2003). Based on human diffusion-tensor imaging studies (Catani et al., 2005; Caspers et al., 2011), we tested whether there was direct baseline connectivity from the PMv to the pSTS, and from the pSTS to the PMv. This allowed the following four patterns: mutual, unidirectional from PMv to pSTS, unidirectional from pSTS to PMv, and no connection (Figure 2B). Then, as modulation effects (B parameters), we assumed that Execution and Hand observation factors modulated all connections involved in baseline connectivity. Finally, we defined three of the direct inputs (C parameters) as follows: the cue for the change detection-task inputs, Execution, and Hand observation. The task cue input to the OP in all of the models. We hypothesized that Execution input to the PMv and Hand observation input to the pSTS. This was based on previous studies suggesting that hand observation activates the pSTS, which codes the visual properties of the consequences of motor execution, and that the PMv codes the action vocabulary (Rizzolatti et al., 1988, 2002; Schubotz and von Cramon, 2004; Fazio et al., 2009). The cue specifying the condition for Execution entered into the occipital cortex, and should effectively reach the PMv. Similarly the cue for Hand Observation should reach the pSTS. To reduce complexity and to allow for meaningful inference, a few key regions involved in the process of interest should be selected. Thus, we replaced the endogenous input from a sub-network we are not interested in with exogenous inputs that approximate the influence from this sub-network (Stephan et al., 2010). This model is considered as a parsimonious representation of other possible models, with additional intermediate or relay regions. The presence or absence of the inputs (Execution to PMv, and Hand observation to pSTS) allowed the following four patterns: dual, single to the PMv, single to the pSTS, or no input. Thus, we defined and compared 16 DCM models (Figure 2B).

*DCM estimation, model selection, and evaluation of effective connectivity.* All of the coupling parameters, including the baseline connections, modulations to the connections, and driving input in the DCM, were derived using Bayesian estimation schema on an individual basis. As subjects can exhibit different models or functional architectures, the random effects (RFX) Bayesian model selection (BMS) technique was adopted (Stephan et al., 2009). This approach accounts for the heterogeneity of the model structure across subjects. It uses hierarchical Bayesian modeling that estimates the parameters of a Dirichlet distribution over the probabilities of all models considered, enabling the computation of the posterior probability of each model given the data of all subjects and the models considered. The results of RFX analysis are reported in terms of the exceedance probability that one model is more likely than any other model (Stephan et al., 2009). The optimal model would be considered to be the one with the largest exceedance probability.

To elaborate this model selection based on individual-model-level inference, we also conducted family-level inference procedure to characterize the effects of attributes of the same model space (Stephan et al., 2009). This type of inference rests on comparing subsets or families of model space, pooling information over all models in these subsets. This effectively removes uncertainty about any aspect of model structure other than the attribute of interest (which defines the partition). The model space of the present study has two attributes: the number and location of direct inputs, and the baseline connectivity between PMv and pSTS. In terms of the number and location of direct inputs, presence or absence of the direct input to PMv and pSTS, generated four families. We computed the exceedance probability that indicates how likely one specific model family is compared with other families, regardless of any other differences among the models considered. Similar inference was made with another partitioning by means of the connection between PMv and pSTS, generating four families (Figure 2). The optimal model in family-level inference would be the one with the largest exceedance probability of both attributes.

In the RFX model framework, coupling parameters of the baseline connections (A parameter estimates), and modulation factors (B parameter estimates) within the best model selected by BMS, are random effects in population. Thus, these subject-specific estimates of the parameters were entered into one-sample *t*-test or paired *t*-test with false-discovery rate (FDR) correction for multiple comparisons (Benjamini and Hochberg, 1995).

## Results

As the preliminary data analysis did not show any effect of the rotation direction of the visually presented balls, the results are reported with this factor collapsed.

### Performance

The accuracy rates in each condition, analyzed with a one-sample *t*-test, were significantly greater than the expected chance level of 33.3% (*p* < 0.05 with Bonferroni correction), confirming that the participants performed well on the task.

### EMG

**Figure 3:**
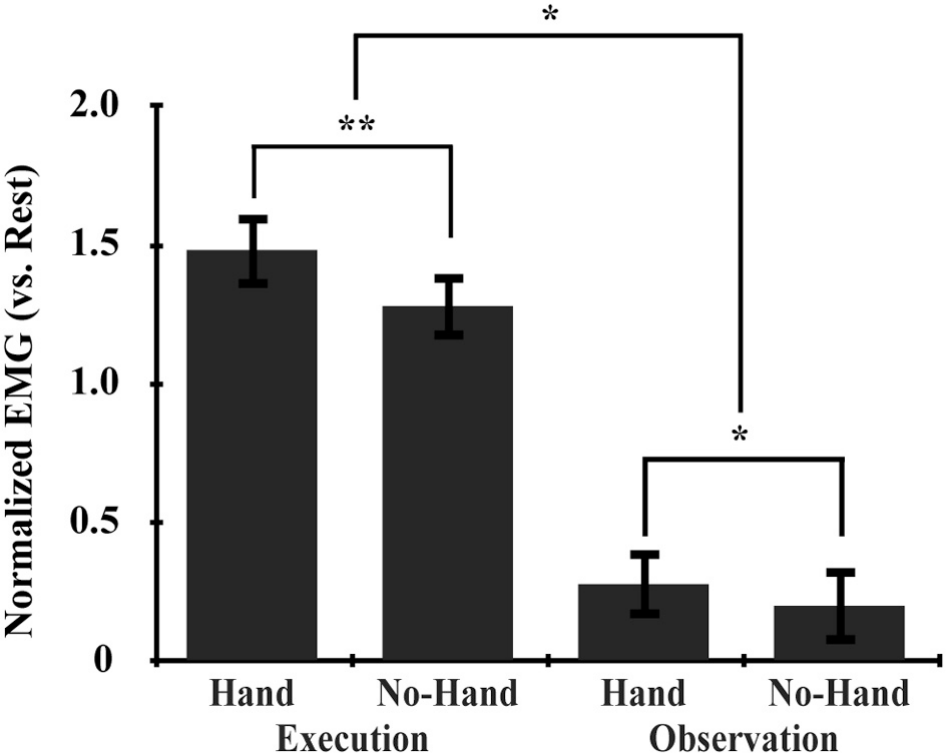
**EMG data.** The normalized EMG value was calculated by subtracting that during the rest condition from that during the task. Asterisks indicate significant differences between conditions with three-way rmANOVA (^**^p < 0.001; ^*^p < 0.01). All error bars indicate the standard error of the mean (SEM).

EMG analysis was performed on the averaged value of two antagonistic muscles: the FCU and the EDC of the right hand (Figure 3). The main effects of Execution and Hand (*p* < 0.001, repeated measures [rm] ANOVA), and their interaction (Execution × Hand) (*p* = 0.001) were statistically significant. *Post-hoc* comparisons showed that the Hand effect was significant during both the Execution (*p* < 0.001) and the Observation (*p* = 0.005) conditions; that is, the EMG signal was greater when observing another's hand movement than when observing ball rotation without a hand. These results indicated that observing the hand movement of others automatically enhanced the EMG signal from the hand, which in turn was enhanced by executing ball rotation, thereby enhancing the excitability of the M1. This suggested that automatic mimicry had occurred.

### fMRI task-related activation

**Figure 4:**
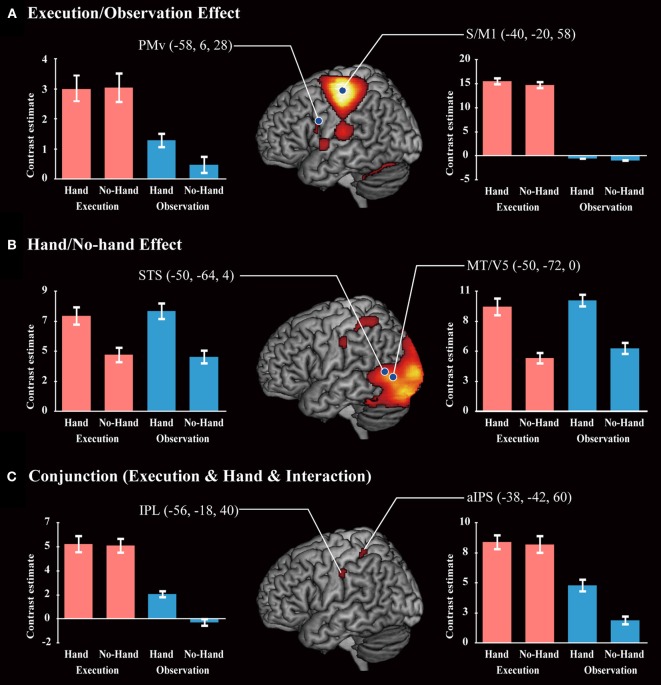
**Brain activity. (A)** A positive Execution/Observation effect was found by the contrast with [Execution > Observation]. **(B)** A positive Hand/No-hand effect was found by the respective contrasts with [Hand > No-hand]. **(C)** A conjunction result was found by the contrast of [Execution > Observation] and [Hand > No-hand] and [Interaction: (Execution > Observation) × (Hand > No-hand)]. All results were thresholded at *p* < 0.05 (FWE). Red bars and blue bars show the data for the execution and observation conditions, respectively. Blue dots on the brain images indicate the locations of the ROIs for DCM analysis.

A main effect of Execution was found in the S/M1, SMA, IPL, aIPS, IFG, and thalamus of the left hemisphere. The insula, dorsal premotor cortex, and postcentral gyrus of the right hemisphere also showed an Execution effect. The bilateral PMv, Rolandic operculum (equivalent to the secondary somatosensory area), and cerebellum were also activated (Figure 4A and Table 4). A Hand effect was found in the visual areas including the MT/V5 and pSTS region bilaterally. The bilateral aIPS, superior parietal lobule, postcentral gyrus, and hippocampus also showed a Hand effect. In addition, the superior temporal gyrus (STG), IPL, and PMv in the left hemisphere, and the right precuneus, were activated (Figure 4B and Table 5). The conjunction analysis showed common activation by the [Execution vs. Observation], [Hand vs. No-hand], and [Execution × Hand interaction] contrasts in the left aIPS and left IPL (*p* < 0.05 with FWE correction at the voxel level; Figure 4C and Table 6).

**Table 4:** **Execution effect**.

**Cluster**	**Cluster**	**Voxel**	**Voxel**					
***P***	**Size**	***P***	***T***	***x***	***y***	***z***	**Side**	**Location**
<0.001	7533	<0.001	29.17	−40	−20	58	L	S/M1
		<0.001	12.44	−4	−2	52	L	SMA
		<0.001	12.04	−16	−20	4	L	Thalamus
		<0.001	9.56	−56	−18	40	L	IPL
		<0.001	6.97	−38	−42	60	L	aIPS
<0.001	152	<0.001	6.5	36	−6	62	R	PMd
<0.001	121	<0.001	6.14	58	−18	48	R	PoCG
0.025	5	0.027	4.86	60	8	32	R	PMv
0.008	23	0.012	5.07	−58	6	28	L	PMv
		0.02	4.94	−60	8	18	L	IFG
0.025	5	0.038	4.77	56	−16	16	R	Rolandic operculum
<0.001	270	<0.001	8.15	−46	0	6	L	Rolandic operculum
0.008	23	0.006	5.22	44	2	6	R	Insula
<0.001	3882	<0.001	30.08	20	−54	−26	R	Cerebellum
		<0.001	14.55	6	−66	−20	R	Cerebellar vermis
<0.001	1018	<0.001	13.09	−22	−56	−24	L	Cerebellum

aIPS, anterior intraparietal sulcus; IFG, inferior frontal gyrus; IPL, inferior parietal lobule; PMd, dorsal premotor; PMv, ventral premotor cortex; PoCG, postcentral gyrus; S/M1, primary sensorimotor cortex; SMA, supplementary motor area. Location was defined using the Anatomy Toolbox (Eickhoff et al., 2005). p-values were corrected for multiple comparisons at the cluster or voxel level with the search volume of the entire brain.

**Table 5:** **Hand effect**.

**Cluster**	**Cluster**	**Voxel**	**Voxel**					
***P***	**Size**	***P***	***T***	***x***	***y***	***z***	**Side**	**Location**
<0.001	14187	<0.001	21.45	28	−96	2	R	MOG
		<0.001	18.54	−24	−88	−16	L	LG
		<0.001	18.41	−24	−98	8	L	MOG
		<0.001	15.34	−50	−72	0	L	MT/V5
		<0.001	11.96	−50	−64	4	L	pSTS
0.035	2	0.024	4.88	18	−54	70	R	SPL
<0.001	367	<0.001	6.84	−32	−48	58	L	SPL
		0.002	5.5	−36	−32	48	L	PoCG
		0.006	5.22	−38	−42	60	L	aIPS
0.011	17	0.02	4.94	14	−48	18	R	Precuneus
<0.001	632	<0.001	7.44	36	−38	56	R	aIPS
		<0.001	5.83	30	−52	60	R	SPL
		0.011	5.1	32	−40	70	R	PoCG
0.025	5	0.036	4.78	−46	−36	22	L	STG
<0.001	126	<0.001	6.59	−22	−32	−2	L	Hipp
<0.001	288	<0.001	7.91	24	−30	−2	R	Hipp
0.001	68	0.001	5.7	−52	−20	38	L	IPL
0.031	3	0.037	4.77	−60	0	32	L	PMv

aIPS, anterior intraparietal sulcus; Hipp, hippocampus; IPL, inferior parietal lobule; LG, lingual gurus; MOG, middle occipital gyrus; PMv, ventral premotor; PoCG, post central gurus; pSTS, posterior part of superior temporal sulcus; STG, superior temporal gyrus; SPL, superior parietal lobule. Location was defined with the Anatomy Toolbox (Eickhoff et al., 2005). p-values were corrected for multiple comparisons at the cluster or voxel level with the search volume of the entire brain.

**Table 6:** **Conjunction of Execution and Hand effects, and their interaction**.

**Cluster**	**Cluster**	**Voxel**	**Voxel**					
***P***	**Size**	***P***	***T***	***x***	***y***	***z***	**Side**	**Location**
0.008	22	0.004	5.15	−56	−18	40	L	IPL
0.011	16	0.008	5.16	−38	−42	60	L	aIPS

aIPS, anterior intraparietal sulcus; IPL, inferior parietal lobule. p-values for interaction contrast were p-values for Execution × Hand interaction contrast. Location was defined using the Anatomy Toolbox (Eickhoff et al., 2005). p-values were corrected for multiple comparisons at the cluster or voxel level with the search volume of the entire brain.

### fMRI effective connectivity

The BMS method determines the probability of generating one model relative to another as the exceedance probability value, which indicates how likely one model is compared with any other given the data. BMS showed that the exceedance probability value of Model-1 (48.2%) was higher than those of the others (24.0%) for Model 5, the second best (Figure 2C). Model space partitioning by the patterns of direct input revealed that exceedance probability of the model family with two inputs (Execution to PMv and Hand observation to pSTS) was the highest (PMv and pSTS, 95.5%; PMv, 0.8%; pSTS, 3.7%; none, 0.04%; Figure 2B). Similarly, another partitioning by the connection between PMv and pSTS showed that exceedance probability of the model family with mutual connection was the highest (mutual, 71.5%; PMv to pSTS, 20.7%; pSTS to PMv, 7.3%; none, 0.5%; New Figure 2B). These findings indicate that we can be 95.5% confident that the models with two inputs have a greater posterior probability than any other model families. Similarly, we can be 71.5% confident that the model family with mutual connection between PMv and pSTS than any other model families. Thus, the most feasible model should have (1) two inputs (Execution to PMv and Hand observation to pSTS), and (2) mutual connection between PMv and pSTS, that is, the model 1. We selected the Model-1 for the following analysis of coupling parameter.

**Figure 5:**
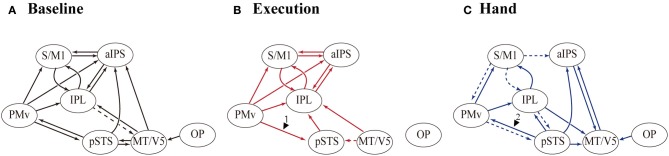
**Effective connectivity evaluated by DCM.** The paths of effective connectivity shown are significantly larger (solid arrows) or smaller (dashed arrows) than 0 (one-sample *t*-test with FDR correction). **(A)** Significant baseline connectivity. Effective connectivity significantly modulated by motor execution **(B)** and by hand observation **(C)** are shown.

**Figure 6:**
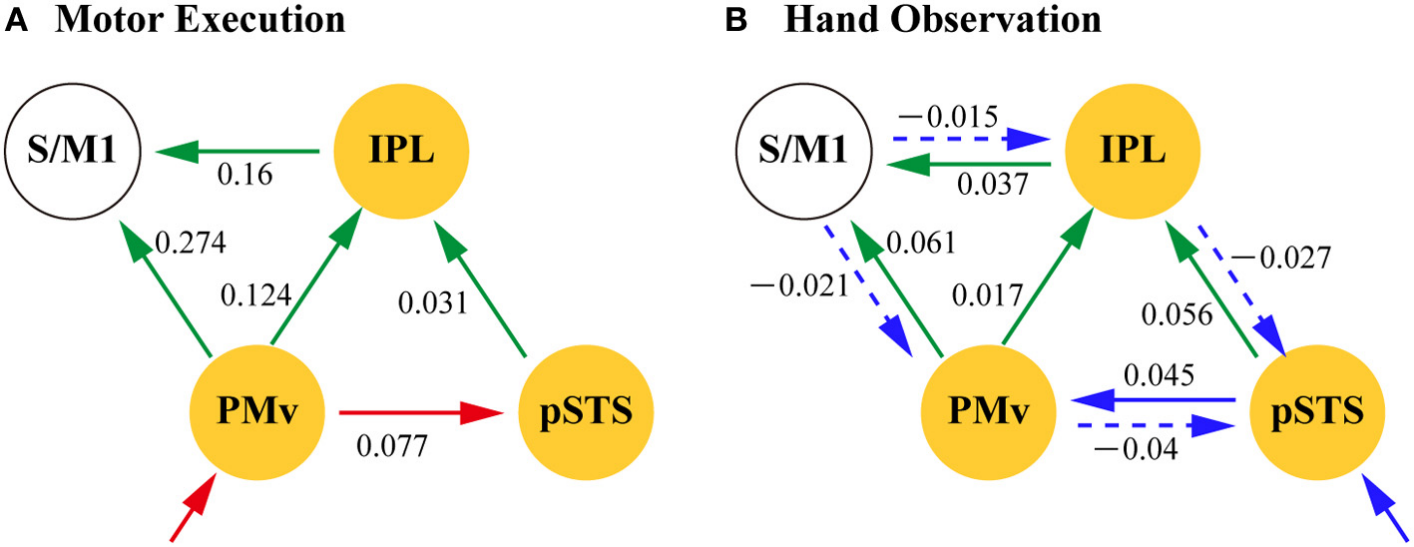
**Dynamic modulation of action representation network by motor execution (A) and Hand Observation (B).** Network nodes (yellow) are the part of the pMNS (putative mirror neuron system). Arrows indicate significant modulation of the effective connectivity by motor execution (red), hand observation (blue), or both (green). Solid arrows indicate positive modulation and broken arrows negative modulation. The values are the group averaged estimation of the modulation of the effective connectivity (Hz). IPL, inferior parietal lobule; pSTS, posterior part of superior temporal sulcus; PMv, ventral premotor; S/M1, primary sensorimotor cortex.

Table 7 summarizes the average coupling parameters of each baseline connection (A parameter estimates). Significant connections constitute the network, as shown in Figure 5A. The results of motor execution modulation (B parameter estimates) are summarized in Table 8. Significantly enhanced connectivity was found among motor-related areas (i.e., the S/M1, PMv, IPL, and aIPS), and from the MT/V5 to the IPL, the pSTS to the IPL, and the PMv to the pSTS (Figure 5B). The results of hand observation modulation (B parameter estimates) are summarized in Table 9. Significantly enhanced connectivity was found among sensory-related areas (i.e., the OP, MT/V5, pSTS, aIPS, and IPL), from the pSTS to the PMv, and from the IPL and the PMv to the S/M1. Suppression of the connectivity was observed from the PMv to the pSTS, and from the S/M1 to the aIPS, IPL, and PMv (Figure 5C). The connections that were commonly modulated by execution and hand observation were observed in the connectivity from the pSTS to the IPL, from the IPL to the S/M1, and from the PMv to the IPL and S/M1 (Figure 6).

**Table 7:** **Results of one-sample *t*-test for coupling parameters of baseline connectivity**.

**BASELINE CONNECTIVITY**								
**FROM**								
		**OP**	**MT/V5**	**pSTS**	**aIPS**	**IPL**	**PMv**	**S/M1**
**TO**								
	MT/V5	**0.387 (0.028)**		**0.169 (0.015)**	−0.018 (0.012)	**−0.036 (0.014)**		
		***p* < 0.001**		***p* < 0.001**	*p* = 0.155	***p* = 0.017**		
	pSTS		**0.115 (0.032)**		−0.003 (0.014)	−0.021 (0.014)	**0.071 (0.020)**	
			***p* = 0.001**		*p* = 0.832	*p* = 0.141	*p* = 0.002	
	aIPS		**0.103 (0.022)**	**0.122 (0.014)**		**0.062 (0.018)**	**0.116 (0.022)**	**0.067 (0.018)**
			***p* < 0.001**	***p* < 0.001**		***p* = 0.002**	***p* < 0.001**	***p* = 0.001**
	IPL		**0.011 (0.024)**	**0.051 (0.012)**	**0.054 (0.021)**		**0.128 (0.019)**	**0.087 (0.024)**
			*p* = 0.658	***p* < 0.001**	***p* = 0.015**		***p* < 0.001**	***p* = 0.002**
	PMv			**0.052 (0.017)**	0.010 (0.014)	−0.008 (0.016)		**−0.004 (0.018)**
				***p* = 0.005**	*p* = 0.504	*p* = 0.635		***p* = 0.003**
	S/M1				**0.053 (0.020)**	**0.121 (0.030)**	**0.229 (0.036)**	
					***p* = 0.016**	***p* = 0.001**	***p* < 0.001**	

The threshold was set at p < 0.026 (corresponding to the FDR corrected p < 0.05). The mean (SEM) and probability are given for each connection. Significant connections are shown in bold. The empty cells represent connections that were not investigated.

**Table 8:** **Results of one-sample *t*-test for coupling parameters of Execution modulation**.

**MODULATION OF EXECUTION**								
**FROM**								
		**OP**	**MT/V5**	**pSTS**	**aIPS**	**IPL**	**PMv**	**S/M1**
**TO**								
	MT/V5	0.034 (0.020)		0.006 (0.010)	−0.012 (0.009)	−0.009 (0.008)		
		*p* = 0.113		*p* = 0.536	*p* = 0.233	*p* = 0.281		
	pSTS		**−0.039 (0.016)**		−0.018 (0.015)	0.007 (0.010)	**0.077 (0.019)**	
			***p* = 0.023**		*p* = 0.231	*p* = 0.470	***p* = 0.001**	
	aIPS		0.022 (0.012)	0.022 (0.014)		**0.039 (0.012)**	**0.119 (0.024)**	**0.055 (0.013)**
			*p* = 0.085	*p* = 0.119		***p* = 0.004**	***p* < 0.001**	***p* < 0.001**
	IPL		**0.030 (0.011)**	**0.031 (0.013)**	**0.037 (0.011)**		**0.124 (0.022)**	**0.069 (0.015)**
			***p* = 0.011**	***p* = 0.0259**	***p* = 0.003**		***p* < 0.001**	***p* < 0.001**
	PMv			−0.018 (0.012)	−0.008 (0.013)	−0.008 (0.014)		0.003 (0.019)
				*p* = 0.154	*p* = 0.565	*p* = 0.592		*p* = 0.856
	S/M1				**0.161 (0.018)**	**0.160 (0.018)**	**0.274 (0.040)**	
					***p* < 0.001**	***p* < 0.001**	***p* < 0.001**	

The threshold was set at p < 0.026 (corresponding to the FDR corrected p < 0.05). The mean (SEM) and probability are given for each connection. Significant connections are shown in bold. The empty cells represent connections that were not investigated.

**Table 9:** **Results of one-sample *t*-test for coupling parameters of Hand observation modulation**.

**MODULATION OF HAND OBSERVATION**								
**FROM**								
		**OP**	**MT/V5**	**pSTS**	**aIPS**	**IPL**	**PMv**	**S/M1**
**TO**								
	MT/V5	**0.203 (0.018)**		**0.213 (0.020)**	**0.051 (0.011)**	**0.030 (0.009)**		
		***p* < 0.001**		***p* < 0.001**	***p* < 0.001**	***p* = 0.003**		
	pSTS		0.002 (0.010)		−0.016 (0.009)	**−0.027 (0.008)**	**−0.040 (0.010)**	
			*p* = 0.837		*p* = 0.097	***p* = 0.003**	***p* = 0.001**	
	aIPS		**0.050 (0.015)**	**0123 (0.020)**		−0.008 (0.07)	0.013 (0.009)	**−0.029 (0.009)**
			***p* = 0.003**	***p* < 0.001**		*p* = 0.271	*p* = 0.167	*p* = 0.002
	IPL		0.017 (0.010)	**0.056 (0.012)**	0.0002 (0.005)		**0.017 (0.005)**	**−0.015 (0.005)**
			*p* = 0.115	***p* < 0.001**	*p* = 0.971		***p* = 0.003**	***p* = 0.008**
	PMv			**0.045 (0.016)**	0.003 (0.009)	−0.010 (0.008)		**−0.021 (0.009)**
				***p* = 0.008**	*p* = 0.740	*p* = 0.195		***p* = 0.021**
	S/M1				0.002 (0.017)	**0.037 (0.012)**	**0.061 (0.018)**	
					*p* = 0.914	***p* = 0.005**	***p* = 0.002**	

Threshold was set at p < 0.026 (corresponding to the FDR corrected p < 0.05). The mean (SEM) and probability are given for each connection. Significant connections are shown in bold. The empty cells represent connections that were not investigated.

The estimated parameters showed that the connectivity from the pSTS to the PMv was enhanced by hand observation and suppressed by execution, and opposite effects were observed from the PMv to the pSTS (Tables 8 and 9, Figure 6). The effective connectivity from the PMv to the pSTS was more prominently modulated by execution than by hand observation (*t*_(23)_ = 5.178, *p* < 0.001, Table 10). The reverse connection from the pSTS to the PMv showed the opposite pattern, and was more prominently modulated by hand observation than by execution (*t*_(23)_ = −3.143, *p* = 0.005).

**Table 10:** **Results of paired *t*-test for comparison of modulation effects of Execution and Hand observation**.

**EXECUTION vs HAND OBSERVATION**								
**FROM**								
		**OP**	**MT/V5**	**pSTS**	**aIPS**	**IPL**	**PMv**	**S/M1**
**TO**								
	MT/V5	**−6.117**		**−9.367**	**−4.339**	**−3.265**		
		***p* < 0.001**		***p* < 0.001**	***p* < 0.001**	***p* = 0.003**		
	pSTS		−1.786		−0.087	**2.687**	**5.178**	
			*p* = 0.087		*p* = 0.932	***p* = 0.013**	***p* < 0.001**	
	aIPS		−1.422	**−3.680**		**3.138**	**4.425**	**5.265**
			*p* = 0.168	***p* = 0.001**		***p* = 0.005**	***p* < 0.001**	***p* < 0.001**
	IPL		0.782	−1.269	**3.198**		**5.366**	**5.658**
			*p* = 0.442	*p* = 0.217	***p* = 0.004**		***p* < 0.001**	***p* < 0.001**
	PMv			**−3.143**	−0.652	0.149		1.355
				***p* = 0.005**	*p* = 0.521	*p* = 0.883		*p* = 0.189
	S/M1				**7.11**	**6.128**	**6.548**	
					***p* < 0.001**	***p* < 0.001**	***p* < 0.001**	

The threshold was set at p < 0.026 (corresponding to the FDR corrected p < 0.05). The t-value and probability are given for each connection. Positive values indicate that the modulation by Execution was larger than that by Hand observation, and vice versa for negative values. Significant connections are shown in bold. The empty cells represent connections that were not investigated.

The effective connectivities from the PMv to the S/M1, the PMv to the IPL, the IPL to the S/M1, and the pSTS to the IPL were significantly modulated by both Execution and Hand observation (Figure 6). The first three of these connections were modulated more prominently by Execution than by Hand observation, and the last did not show a significant difference (Table 10).

## Discussion

### DCM model selection

The present study was designed to depict the forward and inverse internal models as inter-regional relationships during action execution and perception using DCM. Previous studies have suggested that hand observation activates the pSTS that codes the visual properties of the consequences of motor execution, and that the PMv codes the action vocabulary (Rizzolatti et al., 1988, 2002; Schubotz and von Cramon, 2004; Fazio et al., 2009). Thus, we hypothesized that the PMv and the pSTS had terminal positions in the forward and inverse internal models, respectively. Initially, we tested whether Execution directly affected the activity of the PMv, and whether Hand observation affected that of the pSTS (C parameters). Then, based on previous diffusion tensor-imaging studies (Catani et al., 2005; Rilling et al., 2008), we explicitly tested whether the direct baseline connectivity between the PMv and the pSTS was essential (A parameters). An alternative hypothesis was that their relationship was indirect, via the IPL, based on the anatomical connectivity shown in non-human primate studies (Rizzolatti and Luppino, 2001). The two factors were incorporated when generating the 16 models that formed the model space. Regarding the modulation effect of the Execution and Hand observation (B parameters), we assumed that all baseline connections were modulated. This was because we hypothesized that the perceptual-motor networks as a whole constituted a motoric-perceptual action representation, and that their connectivity would accordingly be more or less sensitive to the perturbation of Hand observation and Execution. We therefore tested whether the modulation by Execution and Hand observation was asymmetrically directed between the PMv and the pSTS in the B-parameter space. Consistent with our hypothesis, the BMS procedure selected the model in which motor execution exerted its effect directly on the PMv and modulated the connectivity, and in which hand observation exerted its effect directly on the pSTS and modulated its connectivity.

### PMv

A recent meta-analysis of human fMRI data suggested that the PMv (BA 6) is a homolog of the macaque area F5 (Morin and Grezes, 2008). Most cells in area F5 respond during the execution of motor acts such as grasping, holding, and tearing, and a proportion also responds to passive somatosensory or visual stimulation in the absence of action (Rizzolatti et al., 1988). Area F5 might therefore represent a motor “vocabulary [by which] proximal and distal movement necessary for reaching, grasping, holding, and bringing food to the mouth are represented” (Rizzolatti et al., 1988). In this context, responses to visual objects or somatosensory stimulation were interpreted as a mechanism for sensory stimulation to access various motor acts (Rizzolatti et al., 1988).

### pSTS

The human STS extend from the anterior pole of the temporal lobe to the posterior aspects of the PPC. The anterior aspects are related to speech perception (Hickok and Poeppel, 2000), the central aspects to face and body perception (Haxby et al., 2000; Campbell et al., 2001; Materna et al., 2008), and the posterior and dorsal aspects to social awareness (Martin and Weisberg, 2003; Saxe et al., 2004; Gobbini et al., 2007; Mitchell, 2008). The human pSTS is thought to be the integration site of two visual-processing streams: dorsal brain areas, such as the human MT complex, which support the encoding of action kinematics; and ventral brain areas, such as the fusiform body area and the extrastriate body area, which are proposed to analyze the underlying body postures (Grossman and Blake, 2002; Beauchamp et al., 2003; Giese and Poggio, 2003; Michels et al., 2005; Thompson et al., 2005). The human pSTS is a probable homolog of the superior temporal polysensory (STPa) area in the macaque (Puce and Perrett, 2003). Cells in the STPa respond to a wide range of biological actions and hand–object interactions (Perrett et al., 1985). The human pSTS has been implicated in action recognition (Blake and Shiffrar, 2007; Adolphs, 2009). The response of the pSTS to biological motion is direction, position, and size invariant (Grossman et al., 2010), suggesting that this area is related to the abstraction of the action into object-centered representation during visual analysis, and therefore to action encoding.

### Effective connectivity between PMv and pSTS

Consistent with our hypothesis, the present study showed that the direct effective connectivity between the PMv and pSTS was dependent on execution/hand observation. As effective connectivity is defined as the influence that one neural system exerts over another (Friston et al., 1994), the modulated connectivity is likely to represent the task-related informational flow (Roebroeck et al., 2005; Tanabe et al., 2011; Makuuchi et al., 2012). Thus, the direct effective connectivity from the PMv to the pSTS that is specifically enhanced by execution represents the forward model, and the connectivity in the opposite direction enhanced by observation represents the inverse model. This is consistent with recent human studies in which virtual lesions of the PMv produced by TMS were reported to reduce sensitivity to biological motion (van Kemenade et al., 2012). The lesion sites that were most strongly associated with deficits in biological perception included both the STS and the premotor cortex (Saygin, 2007). Together, these findings suggest that effective connectivity from the pSTS to the PMv codes perception action representation as an inverse internal model.

### Effective connectivity with the IPL

By contrast, the indirect pathway between the PMv and the pSTS through the IPL did not show a modality-dependent directionality of the effective connectivity. Instead, both execution and hand observation commonly enhanced the effective connectivity from the pSTS to the IPL, and from the PMv to the IPL. As the pSTS codes the visual properties of the consequences of motor execution, they are likely to be transferred through robust anatomical connections to the IPL (Seltzer and Pandya, 1994). Similarly, the effective connectivity from the PMv to the IPL that was enhanced by both hand observation and execution might represent the transfer of motor programs. These findings suggest that the left IPL is an essential node in action representation, consistent with previous reports. The IPL and PMv function jointly during motor control (Deiber et al., 1997). The IPL is also related to the integration of somatosensory and visual information (Caminiti et al., 1996; Rizzolatti et al., 1997; for a review see Wise et al., 1997), motor imagery and pantomime comprehension (Sirigu et al., 1996; Rizzolatti and Matelli, 2003; Jeannerod, 2006; Wheaton and Hallett, 2007). The IPL is therefore important for generating action representation.

### Involvement of the S/M1

Commonly enhanced effective connectivity was also observed from the IPL to the S/M1 and from the PMv to the S/M1. EMG recordings showed that observing hand movement activated EMG signals from the hand, which in turn were enhanced by executing ball rotation (Figure 3), confirming the occurrence of automatic mimicry. These findings suggest that action representation is implemented in the motor system including the S/M1 (Fadiga et al., 2005; Kilner and Frith, 2007).

### Feedback control during hand observation

Within the action-representation network involving the PMv, STS, IPL, and S/M1, hand observation suppressed the posterior information flow toward the pSTS from the S/M1 through the PMv and IPL, whereas it enhanced the anterior information flow toward the PMv and SM1. This action-representation network might therefore act as a dynamic feedback-control system during the observation of others' actions (Figure 6B), thereby preventing automatic mimicry.

### Effective connectivity with the aIPS

Both the execution and the observation of grasping and manipulating two balls activated the left ventral portion of the IPL and the aIPS. However, their relationships with other regions differed in terms of effective connectivity. The effective connectivity from the PMv to the aIPS was significantly enhanced by Execution, but not by Hand. Furthermore, the bi-directional connectivity with the IPL and S/M1 was enhanced by Execution. Hand effect was seen with the pSTS and MT/V5, without any modulation of motor nodes (S/M1, IPL, and PMv). This suggests that the aIPS and IPL make different contributions to action representation, and indicates the importance of evaluating network dynamics in order to understand its neural underpinnings.

Recent DCM analysis of functional MRI data from a task involving hand-shape selection in pantomimed grasping (Makuuchi et al., 2012) showed that the neural representation in the aIPS converged on the PMv where grip selection is represented. Using psychophysiological interaction, Hattori et al. (2009) showed that during the judgment of the graspability of objects, the left aIPS had enhanced functional connectivity to the left PMv; they suggested that the connection from the left aIPS is associated specifically with the automatic flow of information about grasping behavior. Grol et al. (2007) showed differential changes in effective connectivity between the aIPS and the PMv during reaching-to-grasp movements. The coupling between the aIPS and the PMv increased more during the execution of a movement toward a small object compared with a larger one. Grol et al. (2007) suggested that this reflects the increased on-line control required to grasp smaller objects. These findings and the present results are also consistent with the notion based on non-human primate studies that the AIP–PMv circuit is concerned with controlling the grasping parameters involved in prehension movements (Jeannerod et al., 1995). Thus, the aIPS might play a role in on-line monitoring and sensorimotor transformation for grasping.

### Conclusions

Action representation of the hand appeared to be implemented as a dynamic interaction between perception and executive brain networks consisting of the S/M1, PMv, IPL, and pSTS. Specifically, direct effective connectivity from the pSTS to the PMv might represent the inverse internal model that underlies automatic mimicry.

### Conflict of interest statement

The authors declare that the research was conducted in the absence of any commercial or financial relationships that could be construed as a potential conflict of interest.
